# Active immunization against alpha-synuclein ameliorates the degenerative pathology and prevents demyelination in a model of multiple system atrophy

**DOI:** 10.1186/s13024-015-0008-9

**Published:** 2015-03-19

**Authors:** Markus Mandler, Elvira Valera, Edward Rockenstein, Michael Mante, Harald Weninger, Christina Patrick, Anthony Adame, Sabine Schmidhuber, Radmila Santic, Achim Schneeberger, Walter Schmidt, Frank Mattner, Eliezer Masliah

**Affiliations:** AFFiRiS AG, Vienna Biocenter, A-1030 Vienna, Austria; Department of Neurosciences, University of California, San Diego, 9500 Gilman Drive, La Jolla, CA 92093 USA; Department of Pathology, University of California, San Diego, 9500 Gilman Drive, La Jolla, CA 92093 USA

**Keywords:** Multiple system atrophy, Active immunization, Immunotherapy, Alpha-synuclein, AFFITOPE**®**

## Abstract

**Background:**

Multiple system atrophy (MSA) is a neurodegenerative disease characterized by parkinsonism, ataxia and dysautonomia. Histopathologically, the hallmark of MSA is the abnormal accumulation of alpha-synuclein (α-syn) within oligodendroglial cells, leading to neuroinflammation, demyelination and neuronal death. Currently, there is no disease-modifying treatment for MSA. In this sense, we have previously shown that next-generation active vaccination technology with short peptides, AFFITOPEs®, was effective in two transgenic models of synucleinopathies at reducing behavioral deficits, α-syn accumulation and inflammation.

**Results:**

In this manuscript, we used the most effective AFFITOPE® (AFF 1) for immunizing MBP-α-syn transgenic mice, a model of MSA that expresses α-syn in oligodendrocytes. Vaccination with AFF 1 resulted in the production of specific anti-α-syn antibodies that crossed into the central nervous system and recognized α-syn aggregates within glial cells. Active vaccination with AFF 1 resulted in decreased accumulation of α-syn, reduced demyelination in neocortex, striatum and corpus callosum, and reduced neurodegeneration. Clearance of α-syn involved activation of microglia and reduced spreading of α-syn to astroglial cells.

**Conclusions:**

This study further validates the efficacy of vaccination with AFFITOPEs® for ameliorating the neurodegenerative pathology in synucleinopathies.

**Electronic supplementary material:**

The online version of this article (doi:10.1186/s13024-015-0008-9) contains supplementary material, which is available to authorized users.

## Background

Multiple system atrophy (MSA) is a progressive, neurodegenerative disease characterized by parkinsonism resistant to dopamine therapy, ataxia, autonomic dysfunction, and pathological accumulation of α-synuclein (α-syn) [1-4]. MSA differs from other synucleinopathies in that α-syn accumulates not only within neurons and astrocytes, but also within oligodendrocytes in the form of glial cytoplasmic inclusions [5]. This intracellular accumulation of toxic α-syn species leads to degeneration of oligodendroglial cells, loss of trophic support to neurons and subsequent neurodegeneration.

In recent years increasing evidence supports the notion that α-syn is primarily generated by neurons, where it aggregates and gets released to the extracellular environment [6,7]. Extracellular aggregated α-syn would then propagate to other neurons and glial cells in a prion-like fashion [8,9]. However, a recent report of MSA oligodendrocytes also expressing α-syn mRNA [10] suggests that the origin of oligodendroglial α-syn might be both of endogenous nature and the result of propagation from neurons and/or other oligodendroglial cells. Furthermore, propagation and accumulation of α-syn within astrocytes could lead to activation of these cells and subsequent neuroinflammation [11-13]. Therefore, the development of therapeutic interventions/strategies for MSA and related neuropathologies has been focused on reducing α-syn accumulation, increasing α-syn clearance and/or inhibiting α-syn propagation. One of these therapeutic alternatives is immunotherapy.

To date there are no disease-modifying treatments for α-synucleinopathies. The discovery that α-syn oligomers can be secreted [14,15] and propagate extracellularly [16,17] provided a clear rationale for immunotherapy [18]. Humoral immunization against α-syn can occur in one of two forms, active or passive immunity [18]. Active immunization involves stimulating the immune system to produce antibodies against toxic α-syn conformations, while passive immunization involves administering anti-α-syn antibodies to the patient, which confers temporary protection against the disease. Recent preclinical studies have been successful in clearing intraneuronal α-syn aggregates and reducing neuron-to-neuron α-syn propagation by immunotherapy, focusing on stimulating or restoring the ability of the immune system to fight the disease [18-22]. In this sense, Phase 1 clinical trial is currently investigating the use of active immunotherapy with PD01A for Parkinson’s disease (PD), and intravenous immunoglobulins are being used in a Phase 2 clinical trial for MSA.

Recent studies suggest that active immunotherapy increases α-syn clearance and might be a viable therapy for PD, a closely related neurodegenerative disease characterized by extensive α-syn deposition in neurons [19,20]. AFFiRiS has developed novel active immunogens (AFFITOPEs®) that hold the promise of treating these disorders. AFFITOPEs® are short immunogenic peptides that are too short for inducing a T-cell response (autoimmunity) and do not carry the native epitope but rather a sequence that mimics the original epitope [23,24]. This methodology allows for the generation of long term, sustained, more specific, non-cross reacting antibody responses suitable for the treatment of synucleinopathies. The main objective of this study was to evaluate the effects vaccination with the AFFITOPE® proven most effective for PD models on reducing the MSA-like pathology in the MBP-α-syn transgenic (tg) mice [19].

## Results

### Titers and trafficking of AFF 1-induced antibodies into the CNS in MBP-α-syn tg mice

**Figure 1:**
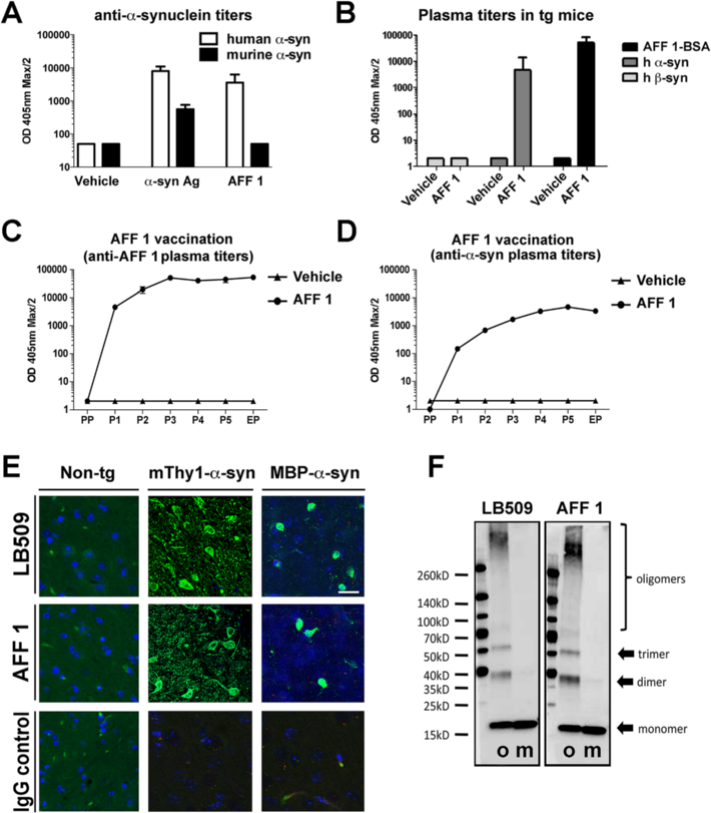
**Titers, kinetics and reactivity of AFF 1-induced antibodies after repeated immunization in MBP-α-syn tg mice. (A)** Titers of antibodies against human and murine α-syn elicited after immunization with vehicle, original C-terminal α-syn antigen or AFF 1 **(B)** IgG response towards the immunizing peptide AFF 1 (as BSA conjugate) as well as against recombinant human α-syn and β-syn from plasma taken at end point **(C)** Kinetics of the IgG responses to the immunizing peptide following vaccination with vehicle or AFF 1 **(D)** Kinetics of the IgG responses to recombinant human α-syn following vaccination with vehicle or AFF 1 **(E)** α-syn immune reactivity of AFF 1-induced antibodies in brain sections of naïve non-tg, mThy1-α-syn tg and MBP-α-syn tg mice. Plasma from AFF 1-immunized MBP-α-syn tg mice was used at a final dilution of 1:100. As positive control, the human α-syn-specific antibody LB509 was used. An anti-mouse IgG antibody was used as negative control. Cell nuclei were stained with DAPI (blue) **(F)** Immunoblot analysis of AFF 1-induced antibodies against aggregated (o) and monomeric (m) recombinant α-syn. The human α-syn-specific antibody LB509 was used as positive control. Aggregated (oligomeric) α-syn was obtained by 4-hydroxy-2-nonenal treatment. Titers are depicted as OD max/2 at 405 nm. PP, Preplasma; EP, end point plasma; P1-P5, plasma taken after each immunization. For titer calculations, n = 10 animals per group.

For the analysis of the immunogenicity and efficacy of AFFITOPE® vaccines in a MSA model, MBP-α-syn tg mice were immunized six times at monthly intervals applying conjugate vaccines containing either the AFFITOPE® AFF 1 (mimicking the C-terminus of α-syn) or the original C-terminal α-syn peptide (α-syn 110–130) coupled to Keyhole limpet hemocyanin (KLH) as carrier and using alhydrogel as adjuvant. As control condition MBP-α-syn tg mice were immunized with the adjuvant alone. Levels of vaccine-induced antibodies were assessed after each immunization (Figure 1A-1D). Both immunogens (AFF 1 and the original C-terminal α-syn peptide) were able to mount a comparable immune response against recombinant human α-syn after three immunizations, thus demonstrating their similar immunogenicities (Figure 1A). In contrast to the original C-terminal α-syn peptide, AFF 1 failed to induce antibodies that cross-react with murine α-syn (Figure 1A). Furthermore, the AFFITOPE® AFF 1 elicited similar antibody titers against the immunizing peptide moiety as the original epitope (Figure 1B and data not shown), but, in contrast to the original α-syn peptide, failed to induce antibodies directed against human β-synuclein (Figure 1B and data not shown).

To study the long-term immunologic responses of AFFITOPE® vaccines in the MBP-α-syn tg model, an analysis of the immune reaction over time was performed assessing reactivity to the immunizing peptide moiety and to human α-syn (Figure 1C, 1D). Titers of antibodies against the peptide moiety of AFF 1 quickly rose after a single immunization (Figure 1C), and reached a plateau after the third immunization. In comparison, titers of antibodies directed against recombinant human α-syn were slightly lower (Figure 1D).

After completion of the immunization protocol, the efficacy of immunization was assessed by histological and biochemical analysis. Immunohistochemical analysis of brain sections from naïve non-tg, mThy1-α-syn tg, and MBP-α-syn tg mice using sera from AFF 1-immunized animals as primary antibody source confirmed that AFF 1-induced antibodies detected intracellular and axonal aggregates in the naïve tg mice but not in the non-tg animals (Figure 1E). AFF 1-induced antibodies displayed reactivity similar to the human α-syn-specific antibody LB509 in this assay. No immunoreactivity was observed with sera of mice immunized with a control vaccine (IgG). To determine which species of α-syn the antibodies elicited by AFF 1 immunization recognize, immunoblot analysis was performed with monomeric and aggregated α-syn (Figure 1F) using 4-hydroxy-2-nonenal [25]. This analysis showed that AFF 1-induced antibodies detected oligomerized α-syn as well as α-syn monomers. The human α-syn-specific antibody LB509 was used as positive control.

**Figure 2:**
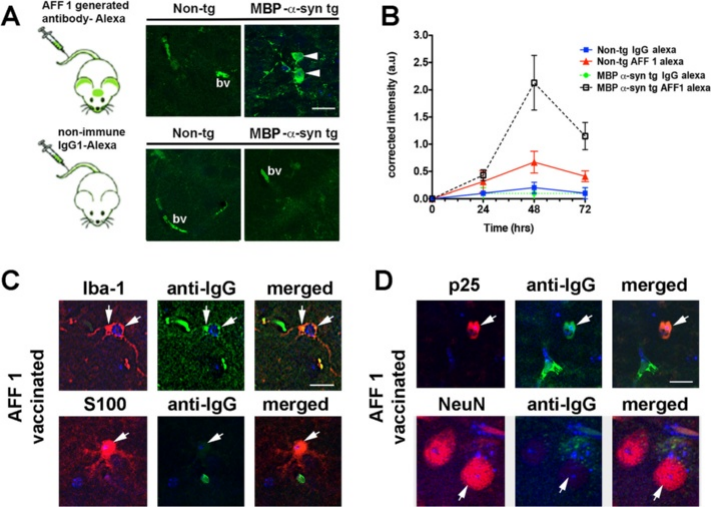
**Trafficking of AFF 1-induced antibodies into the CNS of MBP-α-syn tg mice. (A)** Monoclonal AFF 1-induced antibodies were tagged with Alexa-488 and administered to non-tg or MBP-α-syn mice. Alexa-488 tagged mAb-AFF 1 bounded α-syn within cell bodies (arrow-head) and blood vessels (bv). As negative control, a non-immune Alexa-488-tagged IgG1 was used. Scale bar = 5 μm **(B)** mAb-AFF 1 or non-immune IgG1 were tagged with Alexa-488 and administered to non-tg or MBP-α-syn mice. Time course analysis was performed every 24 h for 3 days, and fluorescence was only increased in brain sections of MBP-α-syn tg animals injected with Alexa-488-tagged mAb-AFF 1. Results are shown as corrected intensity values and expressed as average ± SEM. n = 3 animals per group and time point **(C)** AFF 1-induced antibodies were detected with and FITC-tagged anti-mouse antibody in brain sections of immunized MBP-α-syn tg mice (green), together with an antibody against Iba1 (microglia) or S100 (astrocytes) (red). Cell nuclei were stained with DAPI (blue). Colocalization was observed in microglial cell bodies and projections, but not in astroglial cells (arrows) **(D)** AFF 1-induced antibodies detected with and FITC-tagged anti-mouse antibody in brain sections of immunized MBP-α-syn tg mice (green), together with an antibody against p25 (oligodendrocytes) or NeuN (neurons) (red). Cell nuclei were stained with DAPI (blue). Colocalization was observed in oligodendroglial cell bodies, but not in neurons (arrows). Scale bar = 5 μm.

In order to study the trafficking of AFF 1-induced antibodies into the CNS, a monoclonal antibody derived from an animal undergoing repeated AFF 1 immunization was produced according to standard procedures [26], subsequently tagged with Alexa-488 and injected intravenously into non-tg and MBP-α-syn tg mice (Figure 2A). Vibratome brain sections were analyzed by confocal microscopy 48 h after injection. Only blood vessel labeling was observed in the non-tg mice injected with the Alexa-488-tagged monoclonal antibody mAb-AFF 1. In contrast, the MBP-α-syn tg mice injected with Alexa-488-tagged mAb-AFF 1 showed binding to α-syn aggregates in the neuropil and in cell bodies (Figure 2A), likely after a process of antigen-antibody complex internalization [20,27]. A non-immune IgG1 tagged with Alexa-488 was used as negative control, showing only labeling in blood vessels.

Time-course analysis showed that the highest binding level of the Alexa-488-tagged mAb-AFF 1 was observed after 48 h, with a decline at 72 h (Figure 2C). To assess which cell type internalized the AFF 1-induced antibodies in the MBP-α-syn tg mice, we performed double immunostaining analysis applying an anti-mouse IgG1 and a neuronal (NeuN), astroglial (S100), microglial (Iba1) or oligodendroglial antibody (p25) (Figure 2C, 2D). Anti-mouse antibodies colocalized predominantly with Iba1 in the soma and projections of microglial cells (Figure 2C). Antibodies also colocalized with the oligodendroglial marker p25 (Figure 2D), which indicates that α-syn-expressing cells can also internalize AFFITOPE®-induced antibodies. We did not detected significant colocalization with S100 (Figure 2C), suggesting that antibody-antigen complexes are not significantly internalized by astrocytes. Finally, neurons did not show anti-mouse antibody staining, as detected by double labeling with NeuN (Figure 2D). This result is expected considering the fact that AFF 1-induced antibodies do not react with murine α-syn (Figure 1A) [19].

### Immunization with AFF 1 reduces the accumulation of α-syn aggregates in MBP-α-syn tg mice

**Figure 3:**
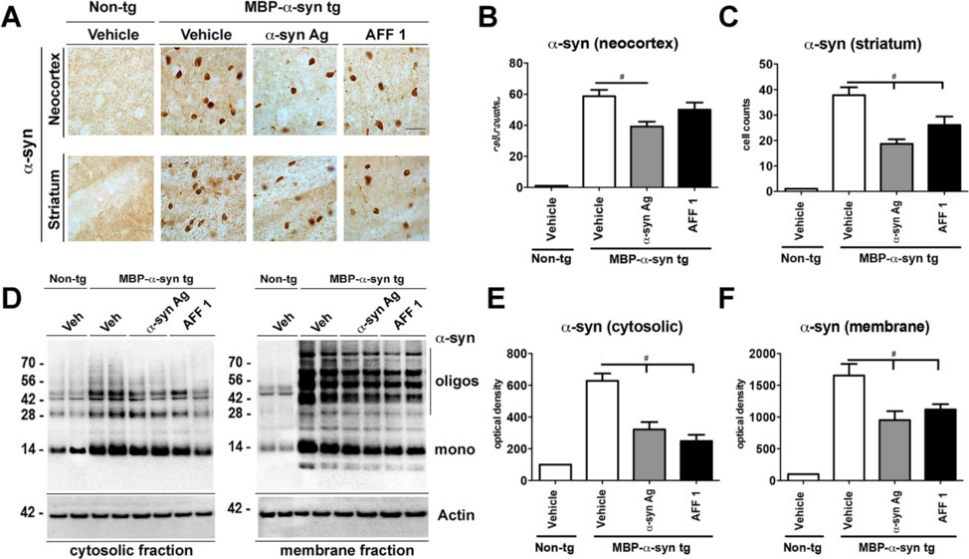
**Immunization with AFF 1 reduces α-syn accumulation in MBP-α-syn tg mice. (A)** α-syn immunoreactivity in neocortex and striatum of non-tg and MBP-α-syn tg mice immunized with vehicle, original C-terminal α-syn antigen, or AFF 1 **(B)** Cell counts of α-syn-positive cells in neocortex **(C)** Cell counts of α-syn-positive cells in striatum **(D)** Immunoblot analysis of total α-syn in the cytosolic and membrane fractions of protein extracts from non-tg and MBP-α-syn tg mice immunized with vehicle, α-syn antigen, or AFF 1. Significant results of two mice per group are shown **(E)** Densitometric analysis of the α-syn immunoreactive bands in the cytosolic fraction **(F)** Densitometric analysis of the α-syn immunoreactive bands in the membrane fraction. Results are expressed as average ± SEM. (#) p < 0.05 when comparing vehicle-treated tg animals vs. immunized tg animals by one-way ANOVA with post hoc Tukey-Kramer. For the vehicle-treated non-tg group, n = 5. For tg groups, n = 10 animals per group.

The α-syn burden was analyzed by immunostaining with an anti-α-syn antibody in the areas most affected by the transgene over-expression, such as the neocortex and striatum (Figure 3A-C). A reduction in the number of α-syn positive cells was observed with both the original α-syn antigen and AFF 1 (Figure 3A), and quantitative analysis revealed a significant reduction in the numbers of α-syn positive cells in both areas following immunotherapy (Figure 3B, C). This reduction in the number of α-syn positive cells was not accompanied by an increase in the number of active caspase 3 positive cells (Additional file 1: Figure S1), suggesting that vaccination is not stimulating the apoptotic death of cells accumulating α-syn, but indeed reducing its accumulation.

Next, the effect of vaccination with AFFITOPEs® on the levels of different α-syn species in the brain was determined by immunoblot. Brain homogenates from MBP-α-syn tg mice immunized with carrier/adjuvant alone (vehicle), original α-syn antigen or AFF 1 were assessed for the content of α-syn monomers and oligomers using an antibody against human α-syn in both soluble and insoluble fractions (Figure 3D-F). Only a slight reduction was observed in the soluble levels of monomeric α-syn (Figure 3D), but a significant reduction was observed in the levels of soluble oligomers (Figure 3D,E). Analysis of the insoluble fraction showed a statistically significant reduction of insoluble α-syn species in the original α-syn antigen and AFF 1-immunized MBP-α-syn tg mice compared to carrier/adjuvant-treated animals (Figure 3D,F). These results confirm that AFF 1 immunization induces antibodies able to specifically bind to and reduce neurotoxic oligomeric/aggregated α-syn.

### Immunization with AFF 1 promotes microglial activation in MBP-α-syn tg mice

**Figure 4:**
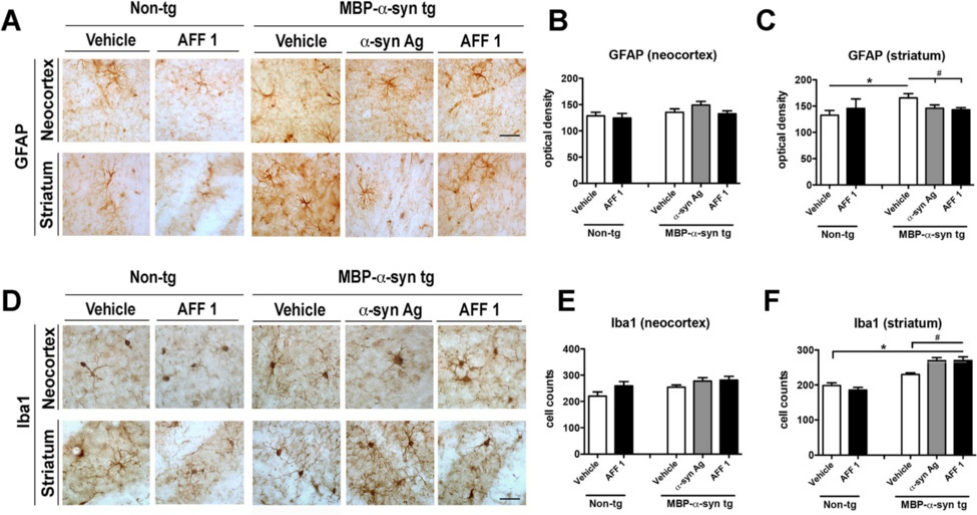
**Immunization with AFF 1 promotes microglial activation in MBP-α-syn tg mice.** Non-tg mice or MBP-α-syn tg mice were immunized with vehicle, original C-terminal α-syn antigen or AFF 1, and glial markers were analyzed by immunohistochemistry. **(A)** Immunostaining of the astroglial marker GFAP in neocortex and striatum. Scale bar = 25 μm **(B)** Optical density quantification of GFAP staining in neocortex **(C)** Optical density quantification of GFAP staining in striatum **(D)** Immunostaining of the microglial marker Iba1 in neocortex and striatum. Scale bar = 25 μm **(E)** Quantification of Iba1-positive cell counts in neocortex **(F)** Quantification of Iba1 cell counts in striatum. Results are expressed as average ± SEM. (*) p < 0.05 when comparing vehicle-treated non-tg animals to tg groups by one-way ANOVA with post hoc Dunnett; (#) p < 0.05 when comparing vehicle-treated tg animals with immunized tg groups by one-way ANOVA with post hoc Tukey-Kramer. For the non-tg group, animal numbers were n = 5 for vehicle and n = 3 for AFF 1. For tg groups, n = 10 animals per group.

In order to analyze if immunization with AFF 1 affects neuroinflammation associated with α-syn accumulation, immunohistochemical analysis of astrogliosis and microgliosis was performed in MBP-α-syn tg mice treated with vehicle, original α-syn antigen or AFF 1 (Figure 4) using the astroglial marker GFAP and the microglial marker Iba1. MBP-α-syn tg mice showed significant astrogliosis and microgliosis when compared to non-tg controls, observed as an apparent increase in the number of cell counts and intensity of the staining in striatum (Figure 4A) and other areas such as hippocampus and corpus callosum (not shown). However we did not observe significant astroglial reactivity in the neocortex of MBP-α-syn tg mice when compared to controls (Figure 4A, B). Immunization with the original α-syn antigen and with AFF 1 significantly reduced astroglial cell counts in striatum to values similar to those observed in non-tg animals (Figure 4C).

Microgliosis was also increased in the striatum of vehicle-treated MBP-α-syn tg mice when compared to vehicle-treated controls, but again not in neocortex (Figure 4D-F). Interestingly, it is worth noticing that MBP-α-syn tg animals do not usually display microgliosis at 10 months/old [28], suggesting that the vehicle (carrier/adjuvant) in combination with the tg phenotype (presence of extracellular α-syn) could be the cause of the microgliosis observed in the striatum of the MBP-α-syn tg animals. Immunization with either the original α-syn antigen or AFF 1 increased microglial cell counts when compared to vehicle-treated tg animals (Figure 4D, F), suggesting that microglial cells are reacting to the presence of induced antibodies and/or antibody-α-syn complexes. In this sense, it is worth noticing that treatment with AFF 1 did not induce changes in microglia cell counts in non-tg animals, indicating that AFF 1 induced antibodies are not inducing microgliosis by themselves but in the form of antibody-α-syn complexes.

**Figure 5:**
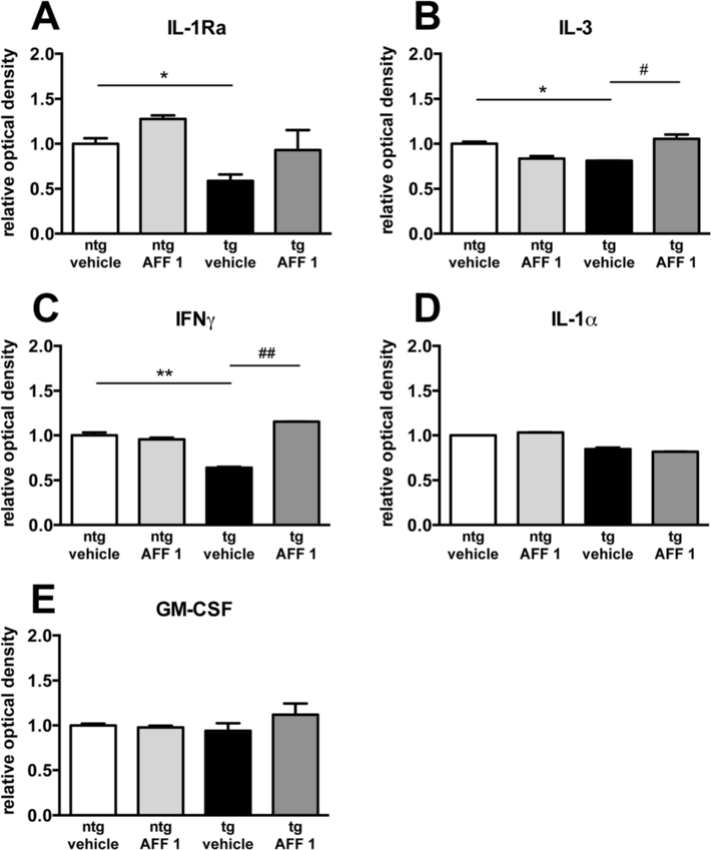
**Immunization with AFF-1 modulates cytokine levels in MBP-α-syn tg mice.** Cytokine levels in the cytosolic fraction of non-tg or MBP-α-syn tg mice treated with vehicle or AFF 1 were analyzed using a proteomic array. Results are expressed as optical density relative to the non-tg vehicle condition. **(A)** IL-1Ra **(B)** IL-3 **(C)** IFNγ **(D)** IL-1α **(E)** GM-CSF. Results are expressed as average ± SEM. (*) p < 0.05, (**) p < 0.01 when comparing vehicle-treated non-tg animals with vehicle-treated tg animals by two-way ANOVA with post hoc Tukey. (#) p < 0.05, (##) p < 0.01 when comparing vehicle-treated tg animals with AFF 1-treated tg animals by two-way ANOVA with post hoc Tukey. n = 4 animals per group.

Accumulation of α-syn in glial cells has been associated to an increase in the expression of pro-inflammatory cytokines [11], and AFF 1 has shown anti-inflammatory properties in an animal model of PD by increasing anti-inflammatory cytokine levels [19]. We analyzed the relative expression levels of 40 cytokines and chemokines in the cytosolic (soluble) fraction of non-tg mice and MBP-α-syn tg mice treated with vehicle or AFF 1 using a mouse cytokine proteomic array, and we observed significant changes in levels of cytokines and chemokines between non-tg and MBP-α-syn tg mice (Figure 5), including a reduction in levels of the anti-inflammatory cytokine IL-1Ra, IL-3 and Interferon γ (IFNγ). IL-1Ra inhibits IL-1α and IL-1β pro-inflammatory signaling by competing with them for receptor binding [29], IL-3 has trophic factor functions in cholinergic neurons [30], and IFNγ plays a dual role in inflammation having both pro- and anti-inflammatory properties [31]. AFF 1 modulated cytokine and chemokine levels, inducing an increase in IL-1Ra, IL-3 and IFNγ to levels similar to non-tg animals. Levels of other cytokines such as GM-CSF and IL-1α, which are traditionally associated with neuroinflammation, were not affected (Figure 5). These results further confirm that immunization with AFF 1 modulates neuroinflammation in MBP-α-syn tg mice.

### Immunization with AFF 1 ameliorates the neurodegenerative pathology in MBP-α-syn tg mice

**Figure 6:**
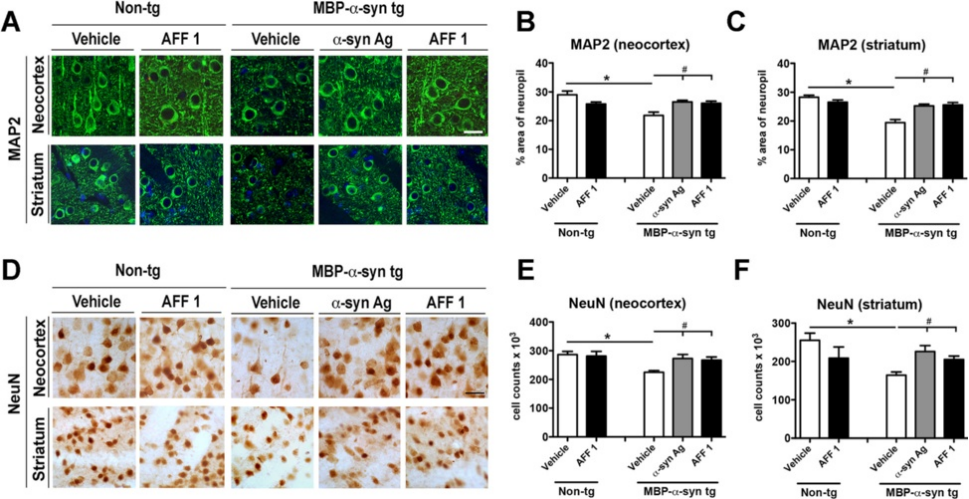
**Immunization with AFF 1 ameliorates the neurodegenerative pathology in MBP-α-syn tg mice. (A)** Immunostaining for the synaptic marker MAP2 (green) in the neocortex and striatum of non-tg mice or MBP-α-syn tg mice immunized with vehicle, original C-terminal α-syn antigen or AFF 1. Cell nuclei were stained with DAPI (blue). Scale bar = 5 μm **(B)** Quantification of the percentage of the MAP2-positive area of neuropil in neocortex **(C)** Quantification of the percentage of the MAP2-positive area of neuropil in striatum **(D)** Immunostaining for the neuronal marker NeuN in the neocortex and striatum of non-tg mice or MBP-α-syn tg mice immunized with vehicle, original C-terminal α-syn antigen or AFF 1. Scale bar = 5 μm **(E)** Quantification of NeuN-positive cell counts per 0.1 mm^3^ in neocortex **(F)** Quantification of NeuN-positive cell counts per 0.1 mm^3^ in striatum. Results are expressed as average ± SEM. (*) p < 0.05 when comparing vehicle-treated non-tg animals to tg groups by one-way ANOVA with post hoc Dunnett; (#) p < 0.05 when comparing vehicle-treated tg animals with immunized tg groups by one-way ANOVA with post hoc Tukey-Kramer. For the non-tg group, animal numbers were n = 5 for vehicle and n = 3 for AFF 1. For tg groups, n = 10 animals per group.

Effects of AFF 1 immunization on synaptic and neurodegenerative pathology were also assessed in MBP-α-syn tg mice. Sections from tg mice treated with vehicle, original α-syn antigen or AFF 1 were immunostained with antibodies against the dendritic marker MAP2, as well as the neuronal marker NeuN (Figure 6A, D). MBP-α-syn tg mice showed a significant decrease in MAP2 staining in the neuropil of neocortex and striatum, which indicates loss of dendritic arborization in these areas (Figure 6A-C). Immunization with both the original α-syn epitope and AFF 1 preserved from the loss of MAP2, and levels in immunized animals were similar to non-tg controls in the affected areas (Figure 6B,C).

MBP-α-syn tg mice are also characterized by the presence of neuronal loss in neocortex and striatum, measured by a reduction in the number of NeuN-positive cells in both areas (Figure 6D-F). This neurodegeneration was also prevented by immunization with both the original α-syn antigen and AFF 1 (Figure 6). Taken together, these results suggest that AFF 1-mediated α-syn clearance is reducing synaptic pathology and neuronal cell death.

### Immunization with AFF 1 reduces demyelination and motor behavioral deficits in MBP-α-syn tg mice

**Figure 7:**
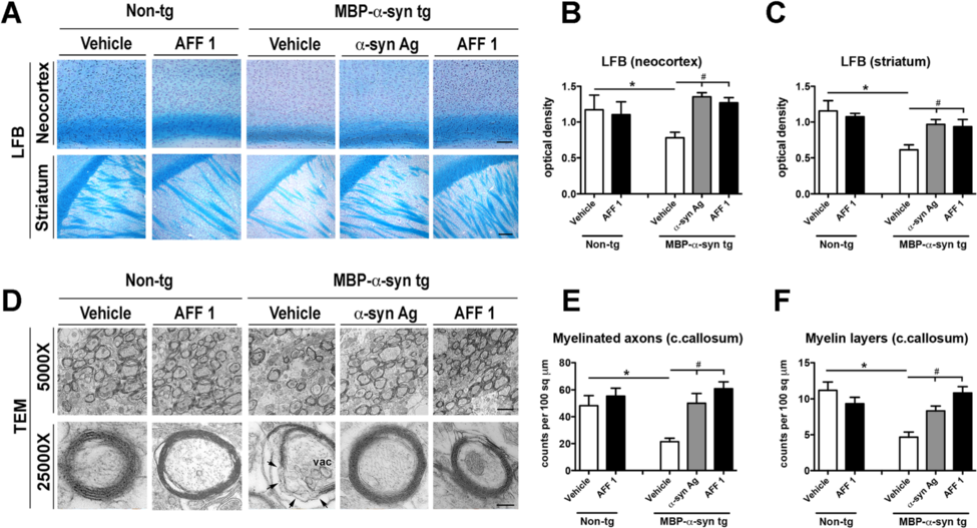
**Immunization with AFF 1 reduces demyelination in MBP-α-syn tg mice. (A)** LFB staining of myelin in the neocortex (and corpus callosum) and striatum of non-tg mice or MBP-α-syn tg mice immunized with vehicle, original C-terminal α-syn antigen or AFF 1. Scale bar = 250 μm **(B)** Quantification of LFB staining by optical density in neocortex **(C)** Quantification of LFB staining by optical density in striatum **(D)** Electron microscopy images of myelin sheaths in the corpus callosum of non-tg mice or MBP-α-syn tg mice immunized either with vehicle, α-syn antigen or AFF 1. Representative images taken with the transmission electron microscope (TEM) are shown at low magnification (5,000x) and high magnification (25,000x). Scale bars = 2.5 μm and 500 nm. vac, vacuola **(E)** Quantification of the number of myelinated axons in corpus callosum **(F)** Quantification of the average number of myelin layers per axon in corpus callosum. Results are expressed as average ± SEM. (*) p < 0.05 when comparing vehicle-treated non-tg animals to tg groups by one-way ANOVA with post hoc Dunnett; (#) p < 0.05 when comparing vehicle-treated tg animals with immunized tg groups by one-way ANOVA with post hoc Tukey-Kramer. For the non-tg group, animal numbers were n = 5 for vehicle and n = 3 for AFF 1. For tg groups, n = 10 animals per group.

To examine the effect of active immunization with AFF 1 on myelination in the MBP-α-syn tg mice, sections were stained with Luxol Fast Blue (LFB) and myelination was analyzed in the neocortex and striatum (Figure 7A-C). Consistent with previous studies in these mice [32,33], the MBP-α-syn tg mice display reduced levels of staining with LFB in comparison to non-tg controls (Figure 7A), indicative of myelin disruption in these mice. Immunization with the original α-syn antigen or AFF 1 reduced demyelination, observed as an increase in LFB staining in both neocortex and striatum (Figure 7A) to values similar to non-tg controls. This reduction in demyelination is likely consequence of the reduction in oligodendroglial α-syn accumulation, and subsequent restoration of myelin production.

Results were further confirmed by visualization of the myelin sheath by electron microscopy in corpus callosum (Figure 7D). In the non-tg mice, the myelin sheath can be observed as a highly organized multilaminar structure (Figure 7D). In the MBP-α-syn tg mice, these structures are less numerous, have less layers and are substantially more disorganized than in non-tg mice (Figure 7D-F). In the MBP-α-syn tg mice immunized with the original α-syn antigen or with AFF 1, the number of myelinated axons as well as the number of myelin layers per axon were preserved and their levels were comparable to non-tg controls (Figure 7E, F). Therefore, it can be concluded that active immunization with AFF 1 reduced demyelinization in the MBP-α-syn tg mouse model of MSA.

**Figure 8:**
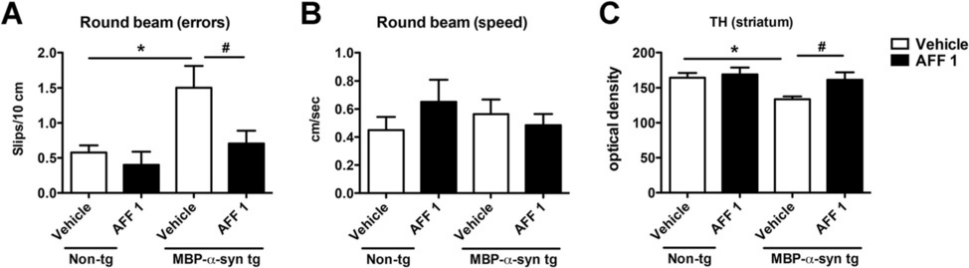
**Immunization with AFF 1 reduces behavioral impairments and TH alterations in MBP-α-syn tg mice. (A)** Performance in the transversal round beam test, measured as slips per 10 cm in non-tg or MBP-α-syn tg mice immunized with vehicle or AFF 1 **(B)** Speed of the animals in the transversal round beam test, measured as cm per second in non-tg or MBP-α-syn tg mice immunized with vehicle or AFF 1 **(C)** Quantification of TH staining by optical density in striatum. Results are expressed as average ± SEM. (*) p < 0.05 when comparing vehicle-treated non-tg animals to vehicle-treated tg animals by one-way ANOVA with post hoc Dunnett; (#) p < 0.05 when comparing vehicle-treated tg animals with AFF 1-treated tg animals by one-way ANOVA with post hoc Tukey-Kramer. For the non-tg group, animal numbers were n = 5 for vehicle and n = 3 for AFF 1. For tg groups, n = 10 animals per group.

In order to investigate if reduced demyelination was accompanied by behavioral improvements in the MBP-α-syn tg mice, vehicle and AFF 1-treated mice were examined on the round beam test to measure gait and balance impairments (Figure 8A, B). Vehicle-treated MBP-α-syn tg mice had a higher error rate (measured as foot slips/10 cm) than vehicle-treated non-tg mice, indicative of balance impairments in the tg animals (Figure 8A). AFF 1 treatment was able to significantly decrease the number or errors made by the MBP-α-syn tg mice (Figure 8A). However, the speed at which the animals performed this task was not significantly altered by the treatment (Figure 8B). Finally, the parkinsonian features have been related to the loss of dopaminergic input to the basal ganglia [34]. In the MBP-α-syn tg mice there is a loss of tyrosine hydroxylase (TH) immunoreactive fibers in the striatum in comparison to the saline-treated non-tg mice (Figure 8C). AFF 1 vaccination restored TH immunoreactivity in the MBP-α-syn tg mice to levels comparable with vehicle-treated non-tg mice (Figure 8C), suggesting a protective effect of the treatment on dopaminergic fiber loss.

### Immunization with AFF 1 increases microglial α-syn clearance in MBP-α-syn tg mice

**Figure 9:**
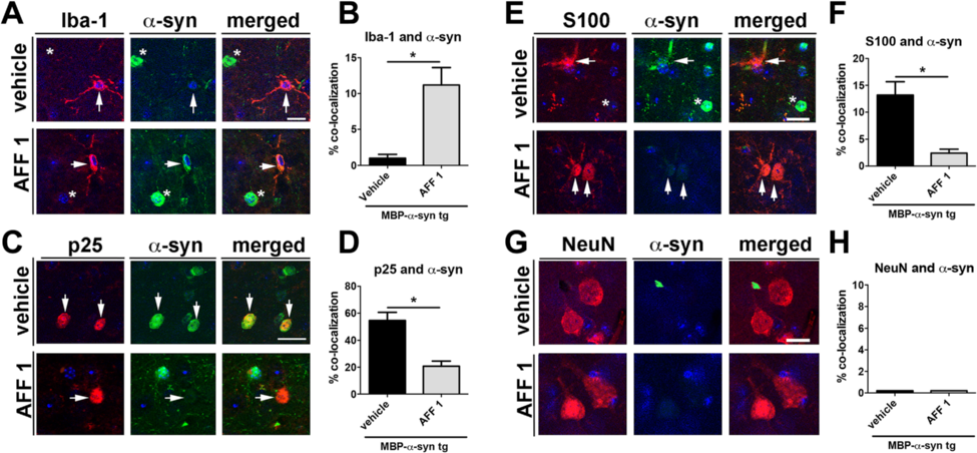
**Immunization with AFF 1 increases microglial α-syn clearance in MBP-α-syn tg mice. (A)** Double immunostaining for the microglial marker Iba1 (red) and α-syn (green) in vehicle- and AFF 1-immunized MBP-α-syn tg mice. The location of Iba1-positive cells is denoted by arrows and the location of α-syn-positive cells by asterisks. Cell nuclei were stained with DAPI (blue). Scale bar = 10 μm. **(B)** Quantification of the percentage of colocalization between Iba1 and α-syn **(C)** Double immunostaining for the oligodendroglial marker p25 (red) and α-syn (green) in vehicle- and AFF 1-immunized MBP-α-syn tg mice. The location of p25-positive cells is denoted by arrows. Cell nuclei were stained with DAPI (blue). Scale bar = 10 μm. **(D)** Quantification of the percentage of colocalization between p25 and α-syn **(E)** Double immunostaining for the astroglial marker S100 (red) and α-syn (green) in vehicle- and AFF 1-immunized MBP-α-syn tg mice. The location of S100-positive cells is denoted by arrows and the location of α-syn-positive cells by asterisks. Cell nuclei were stained with DAPI (blue). Scale bar = 10 μm. **(F)** Quantification of the percentage of colocalization between S100 and α-syn **(G)** Double immunostaining for the neuronal marker NeuN (red) and α-syn (green) in vehicle- and AFF 1-immunized MBP-α-syn tg mice. Cell nuclei were stained with DAPI (blue). Scale bar = 5 μm. **(H)** Quantification of the percentage of colocalization between NeuN and α-syn. Results are expressed as average ± SEM. (*) p < 0.05 when comparing vehicle-treated tg animals with AFF 1-treated tg animals by Student’s *t*-test. n = 10 animals per group.

Finally, to determine which cell type is predominantly involved in antibody-induced α-syn clearance in the MBP-α-syn tg mice, we analyzed the colocalization of neuronal and glial markers with α-syn (Figure 9). As recently shown, microglial cells have the ability to phagocytose α-syn and clear out extracellular α-syn aggregates [35], and astrocytes can also internalize α-syn [11,28]. In MBP-α-syn tg mice, α-syn colocalized predominantly with oligodendrocytes (p25, Figure 9C, D) and with astrocytes (S100, Figure 9E, F). When MBP-α-syn tg mice were immunized with AFF 1 there was a reduction in the percentage of α-syn positive oligodendrocytes and astrocytes (Figure 9D, F) and an increase in colocalization with microglia (Iba1, Figure 9A, B). Importantly, the number of p25-positive oligodendrocytes (25.6 ± 4.2 cells per field) did not change significantly in tg animals after AFF 1 treatment. In a previous study we showed that in MBP-α-syn mice there is propagation of α-syn from oligodendrocytes to astrocytes, and this result suggests that immunotherapy with AFF 1 reduced the extracellular transfer of α-syn to astroglia. Furthermore, the increase in α-syn-positive microglial cells suggests that immunization with AFFITOPEs® is also stimulating the clearance of α-syn by microglial cells. Neurons (NeuN, Figure 9G, H) did not show α-syn staining under any condition.

In conclusion, active immunization with AFF 1 reduces the accumulation of α-syn in oligodendrocytes and astrocytes, prevents demyelination and reduces the neurodegenerative pathology in the MBP-α-syn tg mice, an animal model of MSA.

## Discussion

The present study showed that immunization with the AFFITOPE® vaccine AFF 1 reduced propagation and accumulation of α-syn, demyelination, motor deficits and neurodegeneration in a tg mouse model of MSA. AFF 1 was selected for this study because it had previously demonstrated a high ability to elicit α-syn specific antibodies and to ameliorate behavioral and neurodegenerative pathology in two models of synucleinopathies [19]. Furthermore, AFF 1 elicits the generation of antibodies that do not cross-react with other members of the synuclein family, and does so without promoting α-syn specific T-cell responses [19]. This approach allows for the generation of long term, specific responses suitable for the treatment of synucleinopathies such as MSA.

Active and passive immunization studies have previously shown that immunization reduces the pathology in α-syn tg models of PD. Active immunization of PDGF-α-syn mice with full-length human α-syn induces the production of high affinity antibodies, together with a decrease in neuronal α-syn accumulation and neurodegeneration [20]. Furthermore, vaccination is also effective experimentally in other mouse models of neurodegenerative diseases, by reducing the accumulation of toxic proteins aggregates such as amyloid β [36-39], tau [40,41], prion protein [42] and huntingtin [43,44]. Interestingly, the antibodies produced by mice immunized with full-length human α-syn recognized epitopes within the C-terminal region of human α-syn [20]. Passive immunization studies with antibodies against the C-terminus of α-syn further confirmed this observation [21]. Epitope mapping of AFF 1-induced antibodies shows that the antibodies generated also recognize an area in the C-terminal region of α-syn (110–130) [19], supporting the involvement of this part of the protein in antibody-mediated targeting and clearance. Interestingly, here we report that AFF 1 vaccination also reduced α-syn spreading from oligodendrocytes to astroglia. This finding confirms previous passive immunization studies showing that antibodies against the C-terminus of α-syn also reduced α-syn propagation [45]. Furthermore, this is the first observation that propagation between glial cells can be halted by active immunization. Propagation of α-syn from oligodendrocytes to astroglia has been previously described in the MBP-α-syn mice [28], and although its relevance in MSA has not been determined yet, the recent finding that oligodendrocytes do express α-syn in the MSA brain [10] suggests that this type of propagation could be also happening in MSA. Moreover, reducing propagation of α-syn from oligodendrocytes to astroglia might also prevent neuroinflammatory responses derived from the accumulation of α-syn within astrocytes [11-13]. However, a more detailed analysis of neuroinflammation after immunization with AFF 1 in MBP-α-syn tg mice is needed to elucidate the protective effect of AFF 1 against inflammation in MSA.

First studies on active vaccination against α-syn were performed using full-length human α-syn in the mThy1-α-syn tg model of dementia with Lewy bodies [20]. In this model, active vaccination with human α-syn decreased accumulation of aggregated α-syn in neuronal cell bodies and synapses and reduced neurodegeneration. Antibodies produced by immunized mice recognized abnormal α-syn associated with the neuronal membrane and promoted the degradation of α-syn via lysosomal pathways [20,46]. In the present study we did not observe α-syn or antibody reactivity within neurons, this result suggesting that antibodies elicited by AFF 1 do not recognize murine α-syn, which is expressed predominantly by neurons, and that neurons do not play a significant role in clearing extracellular human α-syn in the MBP-α-syn tg model of MSA. Furthermore, AFF 1 has been previously assessed in an immunization protocol of two different tg mouse models of synucleinopathies, the PDGF-α-syn and the mThy1-α-syn tg mice [19]. Vaccination with AFF 1 in those animals resulted in high antibody titers that crossed into the CNS and recognized α-syn aggregates, similar to the results obtained in MBP-α-syn tg mice. Interestingly in those models clearance of α-syn also involved activation of microglia and increased anti-inflammatory cytokine expression [19]. In this sense and similarly to what we observed in the MBP-α-syn tg mice, AFF 1 also induced an increase the levels of anti-inflammatory cytokines such as IL-1Ra in the PDGF-α-syn tg mouse model of PD [19]. These results combined suggest that the clearance mechanism of extracellular antibody-antigen complexes is probably common to all synucleinopathies. The mechanism through which the antibodies are internalized is not completely understood, but might include interaction with Fcγ receptors [27] and endolysosomal trafficking for autophagy degradation [20]. However, we also observed internalization of antibodies in α-syn-expressing oligodendrocytes in the MBP-α-syn tg mice, suggesting that these cells can also uptake antibody-antigen complexes or that their membrane integrity is compromised and antibodies can access intracellular α-syn deposits. Finally, other clearance mechanisms such as clearing α-syn into the venous system or via other glial mechanisms have not been evaluated and therefore cannot be ruled out.

Also of interest is the fact that immunization with AFF 1 prevented myelin loss and reduced motor deficits in the MBP-α-syn tg mice. MBP-α-syn tg animals show significant myelin pallor in white matter tracts [32], similar to the demyelination observed in the MSA brain [47]. The protective effect of immunotherapy on myelination is probably a consequence of the reduction in α-syn accumulation within oligodendrocytes and/or the reduction in α-syn propagation. Moreover, a direct protective effect of the treatment on myelin integrity cannot be excluded either. Interestingly, this protective effect on myelination has never been reported in active immunization studies for synucleinopathies, and it is a very good indicator of the effects of the AFFITOPE® on the preservation of the brain structure in MSA.

Finally, it is important to consider that MBP-α-syn tg animals over-express human α-syn directly in oligodendrocytes, therefore not showing α-syn propagation from neurons to oligodendrocytes, which it has been reported to occur in MSA patients [48-50]. In this case, AFF 1-induced antibodies might also be beneficial in halting α-syn transmission from neurons [19], which is likely an early event in MSA progression. Moreover, as AFF 1-induced antibodies are specific to human α-syn, the question remains to if they could interact and block the physiological activity of this protein. In this sense, it must be noted that in healthy individuals α-syn should not be present in the extracellular space or bound to the plasma membrane, where the interaction with the antibodies might take place. However, in MSA patients AFF 1-induced antibodies could interact with extracellular or membrane-bound oligomeric α-syn, which are key features of the disease pathology [51]. Therefore, binding of AFF 1-induced antibodies to intracellular, physiological human α-syn is not expected. In this regard, AFF 1 is currently being studied in clinical phase I studies in patients with early MSA.

## Conclusions

In conclusion, targeting extracellular α-syn with active immunotherapy using the AFFITOPE® AFF 1 reduced α-syn accumulation within oligodendrocytes, prevented α-syn spreading and protected myelination in a tg mouse model of MSA. Microglia uptake of antibody antigen complexes is suggested as the clearance mechanism following immunization. Finally, Phase 1 testing is currently in preparation for two AFFITOPE® vaccines that target α-syn using MSA as a model for synucleinopathies.

## Methods

### Generation of AFF 1

The AFFITOPE® AFF 1 was generated and selected as previously described [19]. Briefly, AFF 1 was synthesized by FMOC solid phase peptide synthesis (EMC microcollections GmbH) conjugated to the carrier protein KLH (Biosyn GmbH) using N-gamma-Maleimidobutyryl-oxysuccinimide ester (Thermo Scientific) through an additional N-terminal cysteine residue. AFF 1-KLH conjugates were adsorbed to alhydrogel as adjuvant. The dose used for vaccinating the animals was 30 μg of peptide containing 0.1% alhydrogel.

### Treatment of animals and antibody titers

Mice expressing human α-syn under the control of the Myelin Basic Protein (MBP) promoter (MBP-α-syn tg) were generated as previously described [32]. In this study we used the MBP1 line, as animals express an intermediate level of α-syn compared to the other lines and they are more viable and less aggressive. MBP-α-syn tg mice develop progressive accumulation of α-syn inclusions in oligodendrocytes along the axonal tracts in the brainstem, basal ganglia, cerebellum, corpus callosum, and neocortex, leading to neurodegeneration in the neocortex and to loss of dopaminergic fibers in the basal ganglia. MBP-α-syn tg mice were treated with the original α-syn antigen or AFF 1 plus carrier/adjuvant or carrier/adjuvant alone (n = 10/group). For the non-tg mice, animal numbers were n = 5 for the vehicle-treated group and n = 3 for the AFF 1-treated group. Treatment started when mice were 4–5 months of age, 6 injections were delivered subcutaneously at monthly intervals, and plasma collection was performed two weeks after each injection. Treatment was well tolerated, and no weight loss or other complications were noted. All mice were between 10–11 months of age by the end of the study. All experiments were carried out in accordance with the guidelines laid down by the NIH regarding the care and use of animals for experimental procedures.

Plasma samples from individual animals (n = 10 per condition), obtained two weeks after the third immunization, were subjected to ELISA analysis to measure the titers of antibodies induced by repeated immunization. Substrates used included recombinant human α-syn and β-synuclein (2 μg/ml, rPeptide) and the AFF 1 peptide (EMC microcollections GmbH). The peptides were used as bovine serum albumin (BSA) conjugates (1 μM). Optical density (OD) was measured at 405 nm using a microwell plate reader (Tecan, Switzerland), and titers were expressed as ODmax/2 values.

### Isolation of AFF 1-induced antibodies and labeling

The AFF 1-induced monoclonal antibody (mAb)-AFF 1 (mouse IgG1) directed against α-syn was isolated and labelled with Alexa-488 as previously described [26]. Briefly, BALB/c mice were repeatedly immunized with AFF 1 conjugate vaccine using alhydrogel as adjuvant [26]. Fusion of spleen cells with Ag8.531 myeloma cells and cloning of the hybridoma was performed as previously described [52]. mAb-AFF 1 was purified using a Protein G-sepharose column (HiTrap Sepharose, GE Healthcare) and labelled with Alexa-488 using Alexa Fluor® 488 Protein Labeling Kit (Life Technologies) according to manufacturer’s protocol. For studies of antibody trafficking into the CNS, 6 month-old non-tg and MBP-α-syn tg mice (n = 24/group) were injected intravenously with the Alexa-488-labeled mAb-AFF 1, or a non-immune control Alexa-488-tagged IgG1 at a concentration of 1 mg/kg. Mice were sacrificed at 0, 24, 48 and 72 h after injection (n = 3/group and time point).

### Behavioral testing

MSA is characterized by motor abnormalities such as tremor, rigidity and gait and limb ataxia, and many of these features are replicated in the MBP-α-syn tg mice [32] and reflected in their performance in the round beam test [33]. The round beam test allows for the assessment of gait and balance impairments through distance traveled in an allotted amount of time over a round beam placed horizontally. As previously described [53], three consecutive trials, 1 min each, were run in one day. The total forward distance traveled and the numbers of foot slippages were recorded. Speed on the beam was calculated as distance traveled/time, and errors on the beam were calculated as foot slips/distance traveled.

### Immunoblot analysis

Hemibrains were homogenized and divided into cytosolic and membrane fractions as previously described [54,55]. For immunoblot analysis, 20 μg of total protein per lane was loaded on 4-12% Bis-Tris SDS-PAGE gels and blotted onto polyvinylidene fluoride membranes. To determine the effects of the immunotherapy in levels of α-syn, blotted samples from immunized α-syn tg mice were probed with antibodies against full length human α-syn (1:1000, SYN211, Life Technologies). Incubation with primary antibody was followed by species-appropriate incubation with secondary antibody tagged with horseradish peroxidase (1:5000, Santa Cruz Biotechnology), visualization with enhanced chemiluminescence, and analysis with a Versadoc XL imaging apparatus (BioRad). Analysis of β-actin (Sigma) levels was used as a loading control.

For studying which species the antibodies elicited by AFF 1 recognize, recombinant or 4-hydroxy-2-nonenal-treated α-syn [25] were loaded on 4-12% Bis-Tris SDS-PAGE gels and analyzed by immunoblot using AFF 1-elicited antibodies as primary antibody. The monoclonal antibody LB509 (Covance) served as positive control.

### Mouse cytokine array

400 μg of protein from the cytosolic fraction of brain tissue homogenates (n = 4 per condition) were used for analyzing relative levels of 40 different mouse cytokines using a Mouse cytokine panel array (R&D) following the instructions of the supplier. Briefly, tissue homogenates were diluted and mixed with a cocktail of biotinylated detection antibodies. The mixtures were then incubated with nitrocellulose membranes where capture antibodies are spotted in duplicate for each cytokine. Streptavidin-HRP and chemiluminiscent detection reagents were then added sequentially, and the binding of the detection antibody was detected in a VersaDoc gel-imaging machine (BioRad) and quantified using Quantity One software (BioRad).

### Immunohistochemistry and electron microscopy

At the end of the vaccination protocol, animals were transcardially perfused with physiological saline and brains were collected. Brains were divided sagitally into right and left hemibrains. The left hemibrain was fixed in 4% paraformaldehyde in phosphate buffered saline and serially sectioned in the vibratome, and the right hemibrain was snap-frozen and stored at -80C for subsequent protein extraction. The right hemibrain was serially sectioned with the vibratome at 40 μm and stored at -20C in cryoprotective medium. Sections were immunostained with antibodies against α-syn (Chemicon, 1:250), NeuN (neuronal marker, Millipore, 1:1000), MAP2 (dendrites, Millipore, 1:250), GFAP (astroglial marker, Millipore, 1:500), Iba1 (microglia, Wako, 1:2000), p25 (oligodendrocytes), and horse anti-mouse IgG (Vector Laboratories) and imaged with an Olympus BX54 bright field digital microscope or a laser scanning confocal microscope. For determination of reactivity against α-syn deposits in situ, plasma from AFF 1-vaccinated tg animals was used (dilution 1:100) to stain sections from mThy1-α-syn transgenic and MBP-α-syn transgenic mice. Digital images were analyzed with the ImageQuant 1.43 program (NIH) to determine numbers of α-syn aggregates, dendrites, neurons, astrogliosis and microgliosis [27,33,53]. A minimum of 100 cells was counted per animal and field and counts are expressed as the average number of positive cells per field (230 μm x 184 μm). Stereological analysis of NeuN immunoreactivity was conducted by the dissector method using the Stereo-Investigator System (MBF Bioscience), and the results were normalized to provide cell density information and expressed as cell counts per 0.1 mm^3^. Additional sections were stained with LFB in order to visualize the myelin layers and imaged on the Olympus BX54 brightfield digital microscope. Quantification of LFB staining was performed by obtaining optical density measurements using the ImageQuant software and corrected against background signal levels. Optical density is expressed in arbitrary units. For % area quantifications, threshold values were set to exclusively select neuropil staining and the percentage of the total area was quantified.

For electron microscopy, vibratome sections were postfixed in 1% glutaraldehyde, treated with osmium tetraoxide, embedded in epon araldite and sectioned with the ultramicrotome (Leica). Grids were analyzed with a Zeiss OM 10 electron microscope as previously described [21]. Cells were randomly acquired from 3 grids, and electron micrographs were obtained at a magnification of 5,000X and 25,000X. The number of myelinated axons and myelin layers were quantified in corpus callosum in areas of similar axon size.

### Double immunolabeling

To determine the colocalization between α-syn and neuronal (NeuN) and glial markers (Iba1, S100, p25), double-labeling experiments were performed as previously described [28]. Vibratome sections were immunolabeled with antibodies against S100 (astroglia, Sigma, 1:250), Iba1 (microglia), p25 (oligodendrocytes) or NeuN (neurons), and the immunoreactive structures were detected with the Tyramide Signal Amplification™-Direct system (1:100, NEN Life Sciences), while α-syn was detected with an antibody specific for human α-syn (SYN211, Sigma, 1:250) and a FITC-tagged secondary antibody (Vector Laboratories, 1:75). Cell nuclei were stained using ProLong® Gold Antifade Mountant with DAPI (4′,6-diamidino-2-phenylindole) (Molecular Probes). Sections were imaged with a Zeiss 63X objective on an Axiovert 35 microscope (Zeiss) with an attached MRC1024 laser scanning confocal microscope (BioRad) [56].

### Statistical analysis

Values are expressed as average ± standard error of the mean (SEM). To determine the statistical significance we used one-way analysis of variance (ANOVA) with post-hoc Dunnett test when comparing to the control condition. Additional comparisons were done using Tukey-Kramer post hoc test. For Figure 5, we used two-way ANOVA with post-hoc Tukey. For comparing two groups, Student’s *t*-test was utilized. The differences were considered to be significant if p values were less than 0.05.

## Additional file

Additional file 1: Figure S1. Immunization with AFF 1 does not affect active caspase 3 levels in MBP-α-syn tg mice. Double immunostaining for active caspase 3 (Abcam antibody) (red) and α-syn (green) in vehicle- and AFF 1-immunized MBP-α-syn tg mice. Cell nuclei were stained with DAPI (blue). Negative and positive controls included sections from non-tg naïve mice and from mice treated with kainic acid (not shown). Scale bar = 15 μm.

## Abbreviations

ANOVA: Analysis of variance
α-syn: α-synuclein
BSA: Bovine serum albumin
DAPI: 4′,6-diamidino-2-phenylindole
IgG: Immunoglobulin G
IFNγ: Interferon γ
KLH: Keyhole limpet hemocyanin
LFB: Luxol fast blue
mAb: Monoclonal antibody
MBP: Myelin basic protein
MSA: Multiple system atrophy
OD: Optical density
PD: Parkinson’s disease
SEM: Standard error of the mean
TH: Tyrosine hydroxylase
tg: Transgenic
