# Supplementary figures and images for: Active immunization against alpha-synuclein ameliorates the degenerative pathology and prevents demyelination in a model of multiple system atrophy
Source: Mol Neurodegener. 2015 Mar 19;10:10. doi: 10.1186/s13024-015-0008-9 (PMC4411775; doi:10.1186/s13024-015-0008-9)

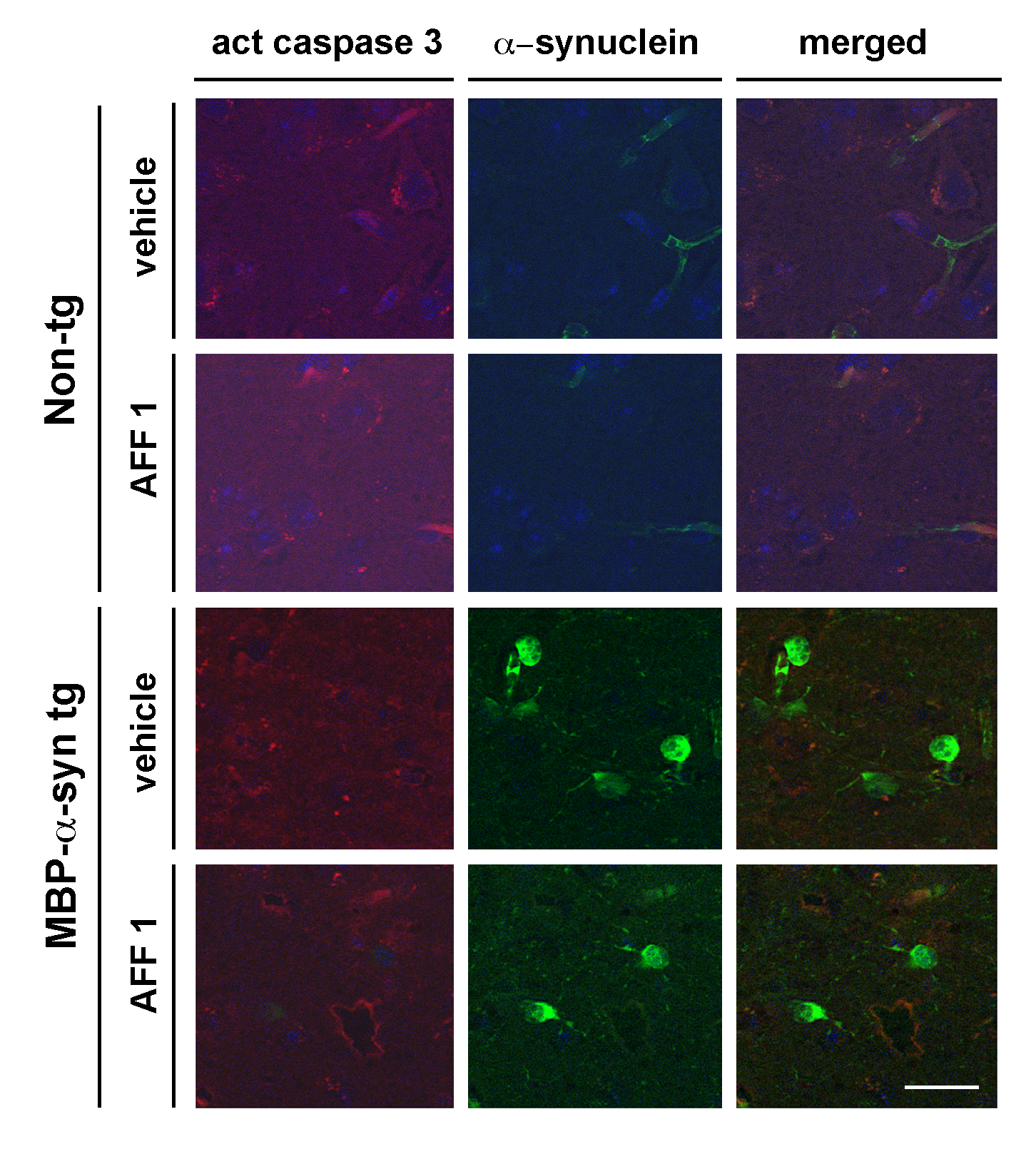

Supplement: Additional file 1: Figure S1. — Immunization with AFF 1 does not affect active caspase 3 levels in MBP-α-syn tg mice. Double immunostaining for active caspase 3 (Abcam antibody) (red) and α-syn (green) in vehicle- and AFF 1-immunized MBP-α-syn tg mice. Cell nuclei were stained with DAPI (blue). Negative and positive controls included sections from non-tg naïve mice and from mice treated with kainic acid (not shown). Scale bar = 15 μm. [file 13024_2015_8_MOESM1_ESM.tiff]
